# Evaluation of the condyle remodeling after lateral arthroplasty in growing children with temporomandibular joint ankylosis

**DOI:** 10.1038/s41598-017-09425-5

**Published:** 2017-08-30

**Authors:** XiuJuan Yang, Chuan Lu, MinJun Dong, DongMei He, Chi Yang, YiHui Hu

**Affiliations:** 1 Department of Oral Surgery, Shanghai Ninth People’s Hospital, College of Stomatology, Shanghai Jiao Tong University School of Medicine, Shanghai, Key Laboratory of Stomatology, Shanghai, 200011 China; 2 Department of Radiology, Ninth People’s Hospital, Shanghai Jiao Tong University School of Medicine, Shanghai, 200011 China

## Abstract

Temporomandibular joint (TMJ) ankylosis in growing patients can cause both mouth opening limitation and jaw bone deformity. Lateral arthroplasty (LAP) can restore the medially displaced condyle and thus keep its growth potential. But can the condyle grow as normal is unknown. This study was to evaluate the long-term result after LAP in growing children. Patients from 2010 to 2014 were evaluated. Their CT data within 1 week after operation and during at least 1-year follow-ups were measured by ProPlan CMF 1.4 software. The condyle-ramus length of both sides and angles and the chin deviation before and after operation were compared. SPSS 17.0 software was used for statistical analysis. A total of 9 patients with a mean age of 10.2 years were included in the study. The mean follow-up period was 1.94 years. The mean condyle-ramus growth was 2.90 mm in the affected side and 2.50 mm in the unaffected side (*P* = 0.31). All of the affected side exhibited growth and remodeling of the condyle and ramus. All 9 cases had a mean chin deviation of 3.69 mm before operation and 2.92 mm during follow-ups (*P* = 0.16). The residual condyle grows after LAP, which can reduce the gravity of jawbone deformity.

## Introduction

Temporomandibular joint (TMJ) ankylosis is a severe maxillofacial malady that can destroy the TMJ structure and restrict the opening of the mouth. It can also induce jawbone deformities. There are few classifications of TMJ ankyloses, one of which was from Sawhney classified four types of ankylosis based on X-ray^1^. Later, in 2008, Yang and He classified ankylosis according to coronal computer tomography (CT), which can be simply divided into two subtypes^2^. In the first subtype, the bony fusion is present only on the lateral aspect of the joint and the residual condylar fragment is present on its medial aspect (same as Sawhney type III). In the second subtype, the bony and/or fibrous fusion is presented through the joint without residual condyle. Different surgical approaches were used according to the subtypes. Regarding the first subtype, we only attempt to remove the lateral bony fusion and keep the medially displaced residual condyle and disc. This procedure was termed lateral arthroplasty (LAP). Regarding the second subtype, it is necessary to resect the bony fusion completely and conduct joint reconstruction. LAP was first proposed by Nitzan^3^, who stated that it may be easy to relapse without filling the lateral space after bony fusion resection^2^. He and Yang reduced the relapse by using a temporalis myofascial flap or a free fat graft from the belly to fill the lateral osteotomic gap^2, 4^. To improve the accuracy of osteotomy and protect the residual condyle, which is hidden behind the bony fusion, skull base, and external auditory canal, a computer-assisted surgical technique including a navigation and osteotomy template were adopted^5, 6^. The long-term results after LAP in growing children were reported by He and Nitzan respectively^4, 7^. However, the amounts of the condylar growth and remodeling in growing children after LAP were not analyzed. Do those patients still need orthognathic surgery to correct jawbone deformity?

In this study, CT data before the operation and during follow-ups at least 1 year after the operation were measured by Proplan CMF 1.4 software (Materialize, Leuven, Belgium) to evaluate the growth and remodeling of condyle-ramus structure in those growing children.

## Results

**Figure 1:**
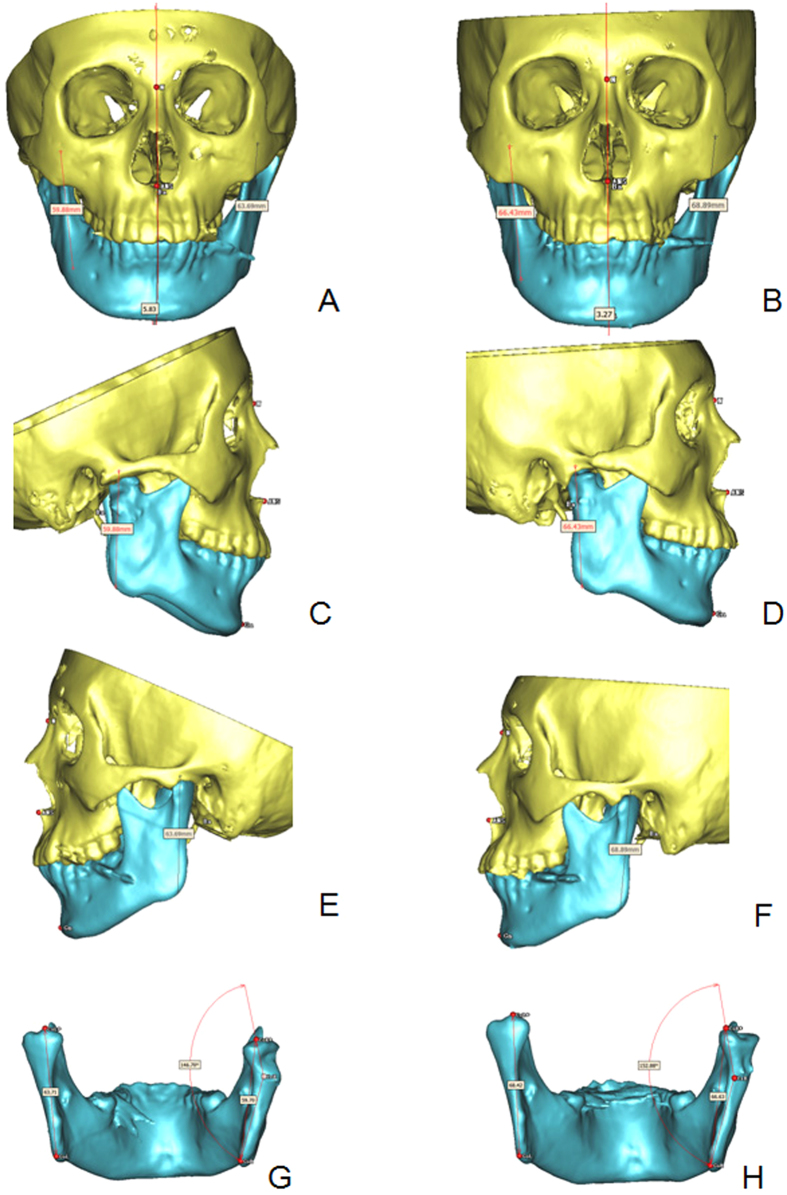
3D measurement of a 13-year-old male (patient 6) before and after operation. (**A**,**C**,**E**,**G**) 1 week after the operation; (**B**,**D**,**F**,**H**) 1.5 years after the operation, the condyle grew and upright remodeled.

**Figure 2:**
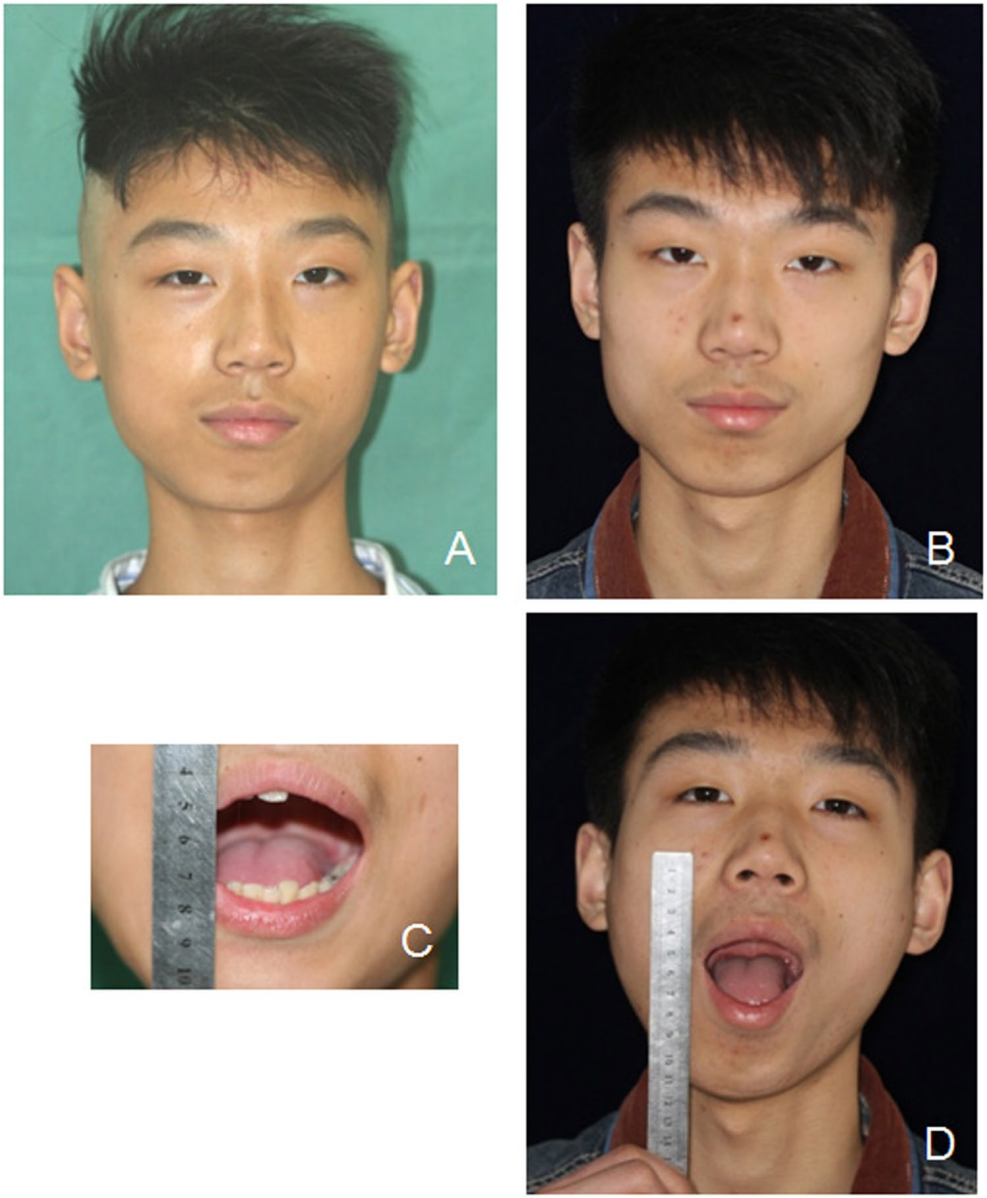
Photographs of the patient in Fig. 1 before and after the operation. (**A**) Preoperative front view; (**B**) 1.5 years postoperative front view; (**C**) Preoperative mouth opening; (**D**) 1.5 years postoperative mouth opening was improved.

**Figure 3:**
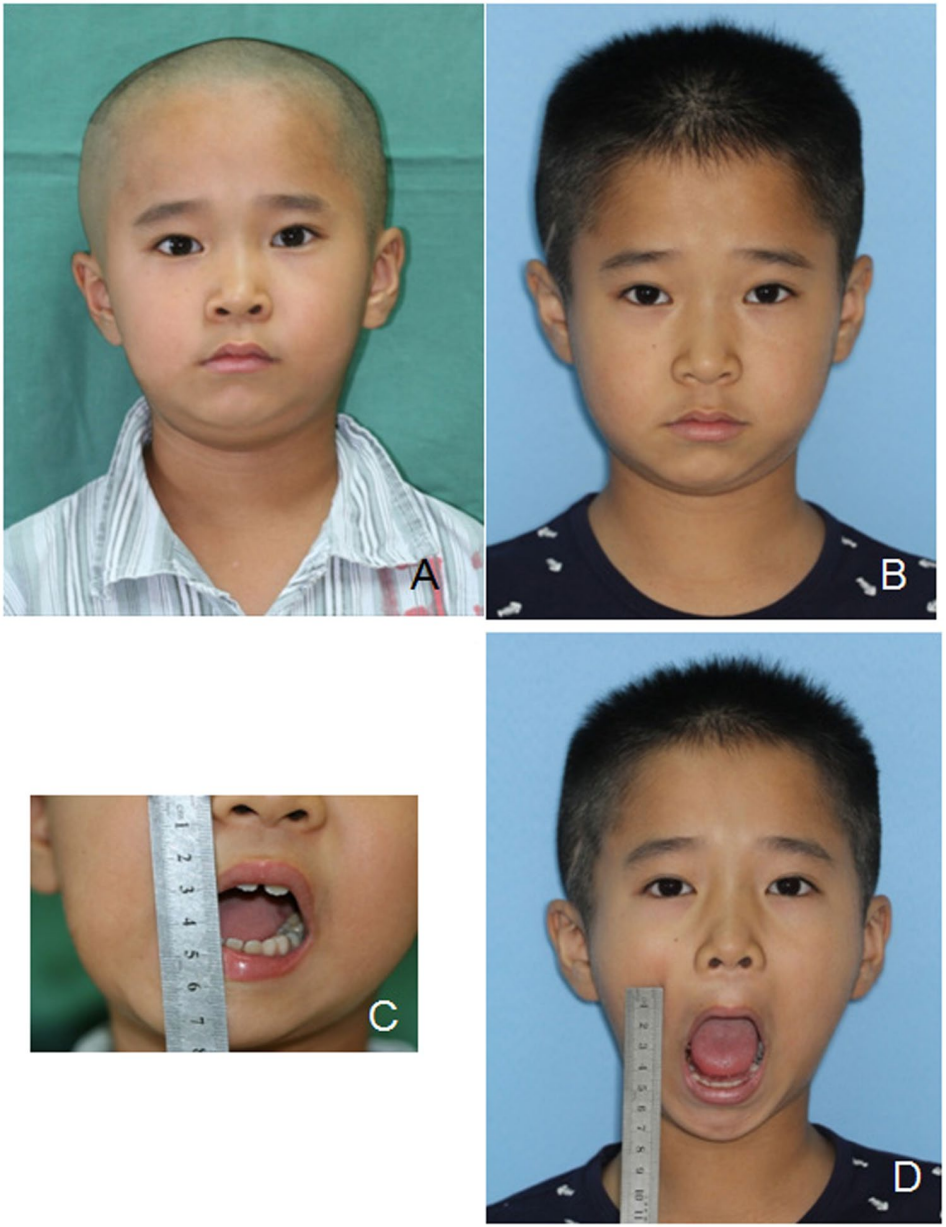
Photographs of an 8-year old boy (patient 3) before and after the operation. (**A**) Preoperative front view; (**B**) 2 years postoperative front view; (**C**) preoperative mouth opening; (**D**) 2 years postoperative mouth opening.

**Figure 4:**
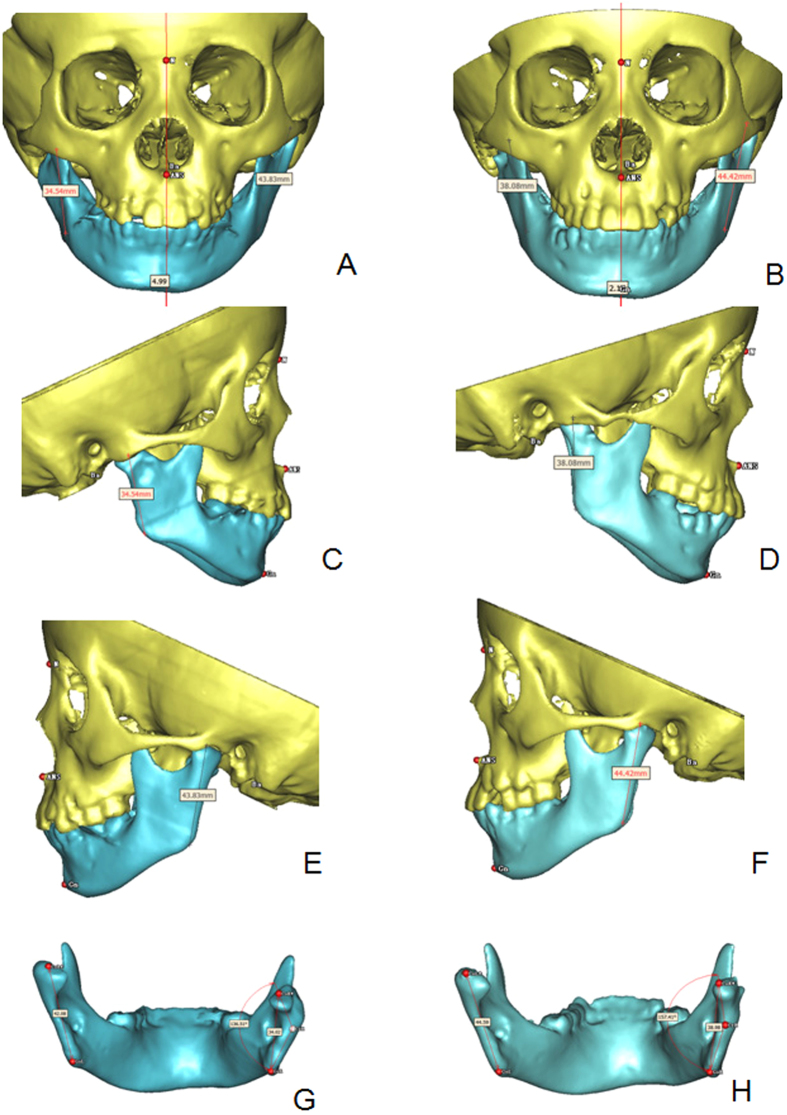
3D CT measurement of the patient in fure 4. (**A**,**C**,**E**,**G**) 1 week after the operation; (**B**,**D**,**F**,**H**) 2 years after the operation, the condyle-ramus grew and upright remodeled.

There were 123 patients diagnosed of TMJ ankylosis and treated from 2010 to 2014. Those patients came to see us at the first time of mouth opening limitation and did not accept any treatment before. Among them, 9 patients were included in the study. Their mean age was 10.2 years old (ranging from 5 to 15 years old). There were 5 males and 4 females. The mean duration of disease was 3.6 years (ranging from 1.5 to 9 years). The mean follow-up period was 1.94 years (ranging from 1 to 3 years). The mouth opening was significantly improved, from 13.22 mm before the operation to 39.44 mm during follow-ups (Table 1, Figs 1–4).

**Table 1:** Information of the growing TMJ ankylosis patients treated by LAP.

Name	Gender	Age (yr)	Duration of disease (yr)	Follow-up (yr)	Mouth opening before surgery (mm)	Mouth opening during follow-ups (mm)
1	F	13	8	2.5	12	28
2	M	5	1.5	3	8	44
3	M	7	4	2	15	43
4	M	11	1.5	3	14	40
5	F	13	9	1	7	35
6	M	13	3	1.5	24	44
7	M	15	2	1	9	44
8	F	9	1.5	2	25	47
9	F	6	2	1.5	5	30
Mean	M (n = 5), F (n = 4)	10.22	3.61	1.94	13.22^*^	39.44^*^

^*^ *P* = 0.000.

All data variables showed normal distribution, so paired t-test was used for comparison of the condyle-ramus growing between the unaffected and affected sides (Table 2). The mean condyle-ramus growth was 2.50 mm in the unaffected side and 2.90 mm in the affected side, with no statistically significant difference (*P* = 0.31). In the unaffected sides, six cases had significant growth (>2 mm), and the other three cases had no significant growth (<2 mm). In the affected sides, seven cases had significant growth and the other two cases had no significant growth.

**Table 2:** Comparison of the condyle-ramus height between the unaffected and affected sides.

Name	Ramus height in the unaffected side		Ramus height in the affected side		Growing condition		
	Post-operation	Follow-up	Post-operation	Follow-up	Healthy side	Affected side	Difference
1	57.6	60.35	51.63	54.29	2.75	2.66	0.09
2	44.33	51.36	39.51	42.36	7.03	2.85	4.18
3	42.08	44.59	34.02	38.98	2.51	4.96	−2.45
4	46.13	47.72	38.58	41.92	1.59	3.34	−1.75
5	48.3	48.02	42.06	40.36	−0.28	−1.7	1.42
6	63.71	68.42	59.70	66.63	4.71	6.93	−2.22
7	56.98	56.55	50.39	51.54	−0.43	1.15	−1.58
8	48.52	50.73	41.38	44.88	2.21	3.5	−1.29
9	40.42	42.8	45.69	48.09	2.38	2.4	−0.02
Mean	49.79	52.28	44.77	47.67	2.50^*^	2.90^*^	−0.40

^*^ *P* = *0.31*.

All of the affected sides exhibited growth and upright remodeling of the condyle. The mean condyle-ramus angle was 147° before the operation and improved to 152° during follow-ups by 3D measurement (Table 3).

**Table 3:** Remodeling of the condyle-ramus morphology.

Condyle-ramus angle	Pre-operation (°)	Postoperative follow-up (°)
1	145	150
2	140	145
3	136	157
4	140	140
5	145	145
6	147	153
7	155	155
8	160	165
9	160	160
Mean	147^*^	152^*^

^#^ *P* = *0.03*.

Nine cases had a mean chin deviation of 3.69 mm before the operation and reduced to 2.92 mm during follow-ups. Although there were no significant difference (*P* = 0.16), two cases of moderate chin deviation changed to mild deviation (Tables 4 and 5).

**Table 4:** Comparison of chin deviation before the operation and during follow-ups.

Name	Pre-operation	Postoperative follow-up	Difference
1	3.08	1.88	−1.2
2	0.16	4.15	3.99
3	4.99	2.12	−2.87
4	1.41	0.37	−1.04
5	8.16	9.03	0.87
6	5.83	3.27	−2.56
7	3.6	1.47	−2.13
8	3.34	3.93	0.59
9	2.64	0.06	−2.58
Mean	3.69^#^	2.92^#^	−0.77

^#^ *P* = *0.16*.

**Table 5:** Comparison of chin deviation degree before the operation and during follow-ups (number of patients).

Degree of deviation	Pre-operation	Postoperative follow-up
<3 mm	3	5
3~6 mm	5	3
>6 mm	1	1

## Discussion

Our previous studies showed that 75% of the TMJ ankylosis had residual condyles (Sawhney type III), which can be saved by LAP without condylar reconstruction^2^. The other 25% of ankylosis maybe caused by infection and trauma, etc which has no residual condyle displaced medially to the bony fusion. It is mainly suitable for TMJ ankyloses caused by condylar fractures. The ability to avoid joint reconstruction is one of the advantages of this technique, along with the fact that the growth potential of the residual condyle can be preserved for young patients during their growing period^8^. When there is no filling of the osteotomic gap, reankylosis can occur easily. He and Yang suggested that using a temporalis myofascial flap to fill the lateral osteotomic gap can effectively reduce reankylosis^2, 4^. It is also very difficult to perform the operation, because the residual condyle hides medially to the ankylotic bone; thus, the surgeon cannot directly visualize the condyle, so it can be injured easily. By studying 3D CT, Nitzan made a further explanation on how to observe and preserve the medially displaced condyles^7^. He and Yang proposed using computer-aided design to determine the dimension of bony fusion, and they manufactured a surgical template to guide osteotomy during the operation^5^. Thus, the bony fusion can be resected completely and the residual condyle can be well preserved. These improved the accuracy and safety of LAP.

Although LAP is an effective surgical technique, how much the condyle can grow and remodel itself has not been reported in growing children. Do patients still need a secondary operation to correct jawbone deformity? Nitzan observed eight growing patients from 6 to 60 months after the operation and found that facial symmetry improved and the residual condyle with its own growth potential can still maintain normal mandibular movement function and occlusion^7^. However, Nitzan’s report was limited to observation of the patients, so it is lacking data based on quantitative measurement. In this study, we used computer-assisted 3D measurement to evaluate the condyle-ramus height and angle at least 1 year after the operation. The results showed normal condyle-ramus growth after bony fusion removal. There was no significant difference between the unaffected side and the affected side. The condyle in the affected side has growth potential and upright remodeling ability after bony fusion removal. This potential was consistent with the healthy side. Therefore, to prevent the aggravation of jawbone deformity, the operation should be performed as soon as possible to relieve joint ankylosis.

The measurement showed that the degree of chin deviation of nine patients during follow-ups decreased compared with the chin deviation of those preoperatively. Although there was no significant difference, the number of the patients with moderate chin deviation reduced from 5 to 3. But one severe chin deviation case did not improve after the operation. This indicated that LAP can recover the condyle-ramus growth in the affected side and prevent a further aggravation of jaw bone deformity. However, for the patients who have severe jawbone deformity preoperatively, it was hard for the affected condyle to grow significantly over the unaffected side. Thus, a further correction of the jawbone deformity should wait until all growing has occurred.

It is generally recognized that both the maxilla and mandible grow most rapidly between the ages 2 and 12. If surgical treatment can be performed during this period, the severity of jawbone deformity can be avoided. Because the mean follow-up period in this study was only 1.94 years, long-term observation is needed for further evaluation. The size of the residual condyle and the condyle-ramus angle may also have a certain influence on its growth and remodeling. Therefore, a larger patient sample and a longer follow-up period are needed.

The evaluation criteria on treatment of ankylosis are restoration of normal motion and function in patients and no ectopic bone formation around the joint to restrict its motion. For growing patient, the ramus should have growth potential. In order to achieve the above goals, complete resection of the bony ankylotic mass laterally around the residual condyle, protect the residual condylar cartilage carefully and use fat graft to eliminate dead space out of the residual condyle are the key points of sucessful treatment. Also release the muscles on the affected side and cut both coronoid process can improve mouth opening when it is not bigger than 30mm during operation. In this study, case 1 and case 9 only got 28 and 30 mm mouth opening during the last follow-up, CT showed no reankylosis. They did not take mouth opening exercise after operation, it maybe the soft tissue scarring limited the mouth opening. So, physical therapy such as mouth opening exercise is highly recommended after operation. All 9 growing patients after LAP in this study had the residual condyle grew and remodelling. Early operation to release bony fusion can avoid the aggravation of jawbone deformity and stimulate condyle growth. However, for patients with severe jawbone deformity, secondary correction of the jaw bone deformity should wait until after all growth has completed.

## Patients and Methods

### Study design

This was a clinical retrospective study of the results in growing TMJ ankylosis patients after lateral arthroplasty. It was approved by the Independent Ethics Committee of the 9th People’s Hospital in March 1^st^, 2016. We confirm that all methods were performed in accordance with the relevant guidelines and regulations. The authors had access to information that could identify individual participants during or after data collection. Patients diagnosed with TMJ ankylosis and treated between January 2010 and December 2014 were recruited for the study. Informed consent was obtained from all patients. Those patients came to see us at the first time of mouth opening limitation and did not accept any treatment before. The criteria for inclusion were: (1) younger than 18 years of age; (2) unilateral TMJ ankylosis; (3) LAP with residual condyle preserved; (4) CT data 1 week after the operation and at least 1 year follow-ups. Patients were excluded if they had (1) bilateral TMJ ankylosis; (2) TMJ replacement and reconstructed with alloplastic or autoplastic materials; (3) 18 years of age or older; (4) lack of CT data.

### Variables

The predictor variable was the condyle-ramus height and angle before the operation versus after the operation and during follow-ups. The outcome variable was the chin deviation.

### Data analysis

Patients’ CT scans (layer thick 0.625 mm) were taken before surgery, within 1 week after the operation, and during follow-ups. The data were imported into ProPlan CMF 1.4 Software (Materialize, Leuven, Belgium) as Dicom and used to perform coronal and 3D reconstruction of the TMJ under bone threshold. Proplan CMF shows measurements with a precision of 0.1 mm/degree following above condition.

### Evaluation of condyle-ramus growth

Condyle-ramus height, angle and chin deviation were measured in the 3D CT reconstruction (Fig. 1). The landmarks are: condylion (top of the condyle) left (CoL), condylion right (CoR); gonion left (GoL), gonion right (GoR), and the condylion ramus point (Cr). The condyle-ramus height was measured as a distance from Co to Go (Co-Go). The angle between the condyle and ramus in the affected side was measured as an angle from Co through Cr to Go. The median sagittal plane was composed of the nasion (N), anterior nasal spine (ANS), and basion (B). Chin deviation was defined as the vertical distance between the gnathion and the median sagittal plane (Gn-Sag).

The condyle-ramus height on both the affected and unaffected sides, the angle of the residual condyle to the ramus and the chin deviation after operation and during follow-ups were measured. The difference in height measurement was defined as no significant change if it was less than 2 mm. The degree of chin deviation was staged as follows: (1) mild (<3 mm); (2) moderate (3–6 mm); and (3) severe (>6 mm).

LAP was performed as previously reported^2, 4, 5^. Free fat from the belly or temporalis myofacial flap was used to fill the surgical defect. Mouth opening exercise was guided one week after operation and last at least 1 year. CT scans were taken within 1 week after the operation to observe the removal of the bony fusion and the residual condyle using coronal and 3D reconstruction. Patients were asked to continue mouth opening training for more than 1 year after the operation. With minimum 1-year follow-ups, the mouth opening and facial pattern of the patients were recorded and compared with those before the operation. CT data were taken and saved during their follow-ups. The size, morphology, growth, and remodeling of the condyles were observed to see if re-ankylosis happens.

### Statistics

By using SPSS 20.0 software, the normality tests of the data were tested. When it showed normal distribution, the condyle-ramus height of both the affected and unaffected sides, the mouth opening, and the condyle-ramus angle of the affected side before the operation and during follow-ups were compared using paired t-test in the Statistical Package for Social Sciences software package, version 17.0 (SPSS, Chicago, IL, USA). One-way analysis of variance (ANOVA) was used and an α level of ≤0.05 was considered significant.
